# Risk factors for acute kidney injury and kidney relapse in patients with lupus podocytopathy

**DOI:** 10.1093/ckj/sfae148

**Published:** 2024-05-10

**Authors:** Wen Xia, Jiayi Deng, Lulu Zhuang, Feng Xu, Ying Jin, Houan Zhou, Ti Zhang, Zhengzhao Liu, Haitao Zhang, Caihong Zeng, Zhihong Liu, Weixin Hu

**Affiliations:** National Clinical Research Center of Kidney Diseases, Jinling Hospital, Affiliated Hospital of Medical School, Nanjing University, Nanjing, China; National Clinical Research Center of Kidney Diseases, Jinling Hospital, Affiliated Hospital of Medical School, Nanjing University, Nanjing, China; National Clinical Research Center of Kidney Diseases, Jinling Hospital, Affiliated Hospital of Medical School, Nanjing University, Nanjing, China; National Clinical Research Center of Kidney Diseases, Jinling Hospital, Affiliated Hospital of Medical School, Nanjing University, Nanjing, China; National Clinical Research Center of Kidney Diseases, Jinling Hospital, Affiliated Hospital of Medical School, Nanjing University, Nanjing, China; National Clinical Research Center of Kidney Diseases, Jinling Hospital, Affiliated Hospital of Medical School, Nanjing University, Nanjing, China; National Clinical Research Center of Kidney Diseases, Jinling Hospital, Affiliated Hospital of Medical School, Nanjing University, Nanjing, China; National Clinical Research Center of Kidney Diseases, Jinling Hospital, Affiliated Hospital of Medical School, Nanjing University, Nanjing, China; National Clinical Research Center of Kidney Diseases, Jinling Hospital, Affiliated Hospital of Medical School, Nanjing University, Nanjing, China; National Clinical Research Center of Kidney Diseases, Jinling Hospital, Affiliated Hospital of Medical School, Nanjing University, Nanjing, China; National Clinical Research Center of Kidney Diseases, Jinling Hospital, Affiliated Hospital of Medical School, Nanjing University, Nanjing, China; National Clinical Research Center of Kidney Diseases, Jinling Hospital, Affiliated Hospital of Medical School, Nanjing University, Nanjing, China

**Keywords:** acute kidney injury, kidney relapse, lupus nephritis, lupus podocytopathy

## Abstract

**Background:**

Patients with lupus podocytopathy show a high incidence of acute kidney injury (AKI) and relapse, but the risk factors and mechanisms were unclear. This study analysed the clinicopathological features and risk factors for AKI and relapse in lupus podocytopathy patients.

**Methods:**

The cohort of lupus podocytopathy was generated by screening the biopsies of patients with lupus nephritis (LN) from 2002 to 2022 and was divided into the mild glomerular lesion (MGL) and focal segmental glomerulosclerosis (FSGS) groups based on glomerular morphological characteristics. The acute (ATI) and chronic (CTI) tubulointerstitial lesions were semi-quantitatively scored. Logistic and Cox regressions were employed to identify the risk factors for AKI and relapse, respectively.

**Results:**

Among 6052 LN cases, 98 (1.6%) were diagnosed as lupus podocytopathy, with 71 in the MGL group and 27 in the FSGS group. All patients presented with nephrotic syndrome and 33 (34.7%) of them had AKI. Seventy-seven (78.6%) patients achieved complete renal response (CRR) within 12 weeks of induction treatment, in which there was no difference in the CRR rate between glucocorticoid monotherapy and combination therapy with glucocorticoids plus immunosuppressants. Compared with the MGL group, patients in the FSGS group had significantly higher incidences of hypertension and haematuria; in addition, they had higher Systemic Lupus Erythematosus Disease Activity Index 2000, ATI and CTI scores but a significantly lower CRR rate. Urinary protein ≥7.0 g/24 h and serum C3 ≤0.750 g/l were independent risk factors for AKI. During a median follow-up of 78 months, 57 cases (60.0%) had relapse and none reached the kidney endpoint. Failure to achieve CRR within 12 weeks, maintenance with glucocorticoid monotherapy and AKI at onset were independent risk factors for kidney relapse.

**Conclusions:**

In this study, histological subtypes of lupus podocytopathy were found to be associated with clinical features and treatment response. In addition, several risk factors associated with AKI occurrence and kidney relapse were identified.

## Graphical Abstract

**Graphical Abstract:**
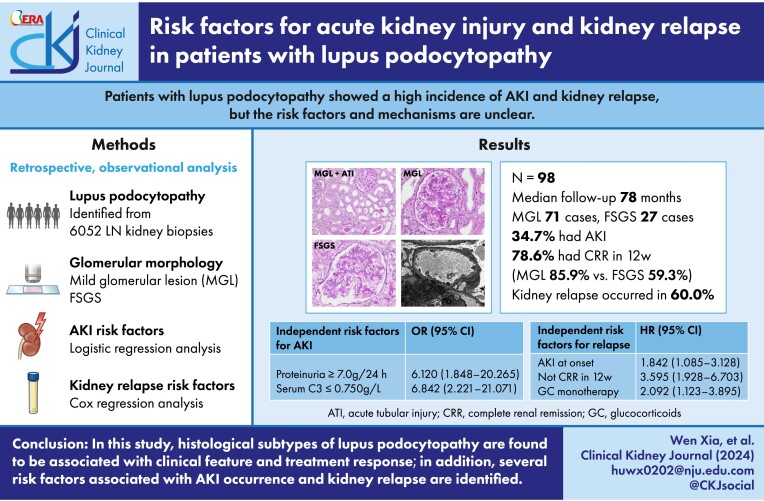

KEY LEARNING POINTS **What was known:**

- Lupus podocytopathy is a novel class of lupus nephritis characterized by extensive foot process effacement without immune deposition in the glomerular capillary loop.
- The high incidence of acute kidney injury (AKI) and the high rate of kidney relapse are challenges in the treatment of lupus podocytopathy, but their risk factors and mechanisms are unclear.

**This study adds:**

- The largest cohort of lupus podocytopathy to date was used, which included 98 cases.
- Patients with lupus podocytopathy and a focal segmental glomerulosclerosis phenotype have a poor treatment response and require optimized treatment regimens.
- High proteinuria and low serum C3 levels are the risk factors for AKI.
- Failure to achieve complete renal remission within 12 weeks, glucocorticoid monotherapy during maintenance and the presence of AKI at onset are the risk factors for kidney relapse.

**Potential impact:**

- This work has provided new insights into the clinicopathological features, treatment response and outcome of lupus podocytopathy.
- Identification of the risk factors for AKI and kidney relapse in patients with lupus podocytopathy will improve treatment decision-making and prognosis evaluation for patients.

## INTRODUCTION

Lupus podocytopathy, a novel form of lupus nephritis (LN), is characterized by podocyte injury mediated by a non-immune complex deposition pathway in patients with systemic lupus erythematosus (SLE) [1–6]. Morphologically, the kidney biopsy exhibits extensive foot process effacement (FPE) of podocytes, with an absence of subendothelial and epithelial electron-dense deposits in the glomerular capillary loop [7, 8].

Previous studies have shown that lupus podocytopathy patients present with nephrotic syndrome (NS) and have a high incidence of acute kidney injury (AKI). They are sensitive to glucocorticoid monotherapy or glucocorticoids combined with other immunosuppressants (combination therapy), but the relapse rate during the maintenance treatment is very high [9, 10]. AKI can result in prolonged hospital stays and increased costs and can affect the course of disease [11, 12], while kidney relapse can increase the accumulated dose of glucocorticoids, the risk for histological transformation, the frequency of hospitalization and economic burden. Given the low incidence of lupus podocytopathy, previous studies have all been limited to a small sample or case report. There has been no study with a large sample size to date analysing the correlation between clinicopathological characteristics and prognosis of lupus podocytopathy, and the risk factors for AKI and kidney relapse are still unclear.

This study aimed to analyse a large-sample cohort of lupus podocytopathy for clinicopathological characteristics and prognosis in order to identify the risk factors for AKI and kidney relapse and assess their impacts on outcome.

## MATERIALS AND METHODS

### Patients

We retrospectively reviewed 6052 LN patients who underwent a kidney biopsy at the National Clinical Research Centre of Kidney Diseases, Jinling Hospital, Nanjing, China from January 2002 to March 2022 to identify those with lupus podocytopathy. Inclusion criteria of lupus podocytopathy were as follows: meeting the diagnostic criteria for SLE of the American College of Rheumatology in 1997; presenting NS and biopsy-proven lupus podocytopathy determined by light microscopy, immunofluorescence and electron microscopy [7] and having complete clinical, laboratory, kidney pathology and follow-up data.

Exclusion criteria included prior use of non-steroidal anti-inflammatory drugs or other medications that might damage podocytes or tubulointerstitium prior to kidney biopsy; a kidney biopsy showing extensive podocyte injury, with LN classes III, IV, V or III/IV + V, or glomerular thrombotic microangiopathy; AKI secondary to kidney vascular lesions, thrombus, urinary obstruction or other abnormalities; and the presence of other glomerular diseases or autoimmune diseases such as anti-neutrophil cytoplasmic antibody–associated small vessel vasculitis, rheumatoid arthritis and scleroderma.

This study was approved by the independent ethics committee of Jinling Hospital (2021NZKY-021-01). Informed consent was obtained from all patients included in this study.

### Kidney pathology

**Figure 1:**
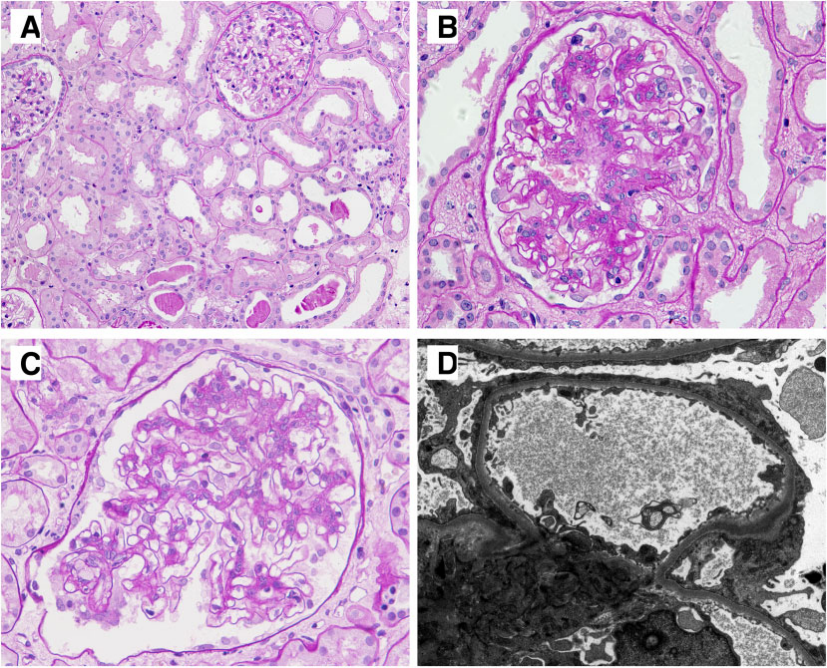
Morphologic features of lupus podocytopathy. **(A)** Glomerular minimal change with acute tubulointerstitial lesions (periodic acid–Schiff, ×200). **(B)** Glomerular mesangial proliferation (periodic acid–Schiff, ×400). **(C)** Glomerular FSGS with a tip lesion (periodic acid–Schiff, ×400). **(D)** Extensive FPE without dense deposits along the glomerular capillary walls (electron microscopy, ×4800).

Lupus podocytopathy was diagnosed based on the following three criteria: the presence of glomerular minimal change disease, mesangial proliferation or focal segmental glomerulosclerosis (FSGS) by light microscopy (Fig. 1A–C); the absence of immune deposits in the glomerular capillary loop with or without immunoglobulin and complement deposits in the mesangial area by immunofluorescence; and diffuse FPE of podocytes (≥50%) in the absence of obvious electron-dense deposits in subendothelial and subepithelial areas (Fig. 1D) as shown by electron microscopy. Based on their glomerular morphology, the patients with lupus podocytopathy were divided into two groups: the mild glomerular lesion (MGL) group (including minimal change disease and mesangial proliferation lesions) and the FSGS group.

LN classes were determined based on revised criteria of the International Society of Nephrology/Renal Pathology Society (ISN/RPS) in 2018 [13]. Semi-quantitative scoring of the acute tubulointerstitial index (ATI) and chronic tubulointerstitial index (CTI) was performed based on the proportion of acute or chronic tubulointerstitial lesions in the whole cortical tubulointerstitial area: 0, no lesion; 1, <25%; 2, 25–49%; and 3, ≥50% or renal tubular necrosis. Acute kidney tubulointerstitial lesions included shedding of the brush border of tubular epithelial cells, epithelial cell necrosis, interstitial oedema or inflammatory infiltration. The chronic tubulointerstitial lesions included tubular atrophy, basement membrane thickening and kidney interstitial fibrosis.

The degree of FPE was calculated by the percentage of FPE in the total length of basement membrane of glomerular capillary loops.

### Clinical and laboratory data

Demographic characteristics, clinical and laboratory data and kidney biopsy results were collected. The Systemic Lupus Erythematosus Disease Activity Index 2000 (SLEDAI-2K) score was used to evaluate SLE activity. NS was defined as proteinuria ≥3.5 g/24 h accompanied by hypoalbuminaemia, oedema and hyperlipidaemia. AKI was defined in accordance with the Kidney Disease: Improving Global Outcomes clinical practice guideline for AKI in 2012 [14]. For those who had no repeated serum creatinine (SCr) assay within 7 days or were recovering from AKI, the diagnostic criteria were expanded to an increase or decrease in SCr of 50% during the hospital stay, using the levels at admission as a baseline [11]. Extrarenal involvement referred to other organ injuries that related to active SLE, excluding the organ injuries caused by infection, medication and other non-lupus activities.

### Treatment regimen and response

Glucocorticoids alone (glucocorticoid monotherapy) or glucocorticoids in combination with other immunosuppressants (combination therapy), including mycophenolate mofetil, tacrolimus, rituximab, azathioprine, leflunomide, cyclophosphamide and tripterygium glycosides (which were extracted from the traditional Chinese herb *Tripterygium wilfordii* and mainly contain triptolide), were used for the initial induction and maintenance therapies. For prednisone induction, a dose of 30–60 mg/d was used for 12 weeks or 2 weeks after complete renal remission (CRR) and then tapered gradually to 10 mg/d for maintenance. Patients were usually seen every 2–4 weeks in the 3 months and then every 1–3 months thereafter. Their treatment response within 12 weeks of induction treatment and the total renal remission rate during the follow-up were evaluated. The treatment response was divided into three categories: CRR, defined by proteinuria <0.5 g/24 h with stable kidney function (SCr ≤10% higher than the baseline levels); partial renal remission (PRR), defined as a decrease in proteinuria of at least 50% and <3.0 g/24 h with stable kidney function (SCr ≤10% higher than the baseline levels); and no remission (NR), defined as failure to achieve CRR or PRR.

Kidney relapse was defined as an increase in urinary protein excretion to ≥1.0 g/24 h for CRR patients and to ≥2.0 g/24 h for PRR patients with or without active urine sediment and an increase of SCr [15]. The kidney endpoint was defined as an estimated glomerular filtration rate ≤15 ml/min/1.73 m^2^ or receiving renal replacement therapy for >3 months. The study endpoint included kidney endpoint, death, kidney histological transformation identified by repeat kidney biopsy after relapse or the end of follow-up (30 October 2022).

### Statistical analysis

Statistical analysis was performed using SPSS version 26.0 (IBM, Armonk, NY, USA). GraphPad Prism version 9.0.0 (GraphPad Software, San Diego, CA, USA) was used for plotting. Continuous variables were presented as median [interquartile range (IQR)], while categorical variables were presented as frequencies (percentages). Independent sample *t*-test (normal distribution) and Mann–Whitney U test (non-normal distribution) were used to compare among groups for continuous variables, as were the Pearson chi-squared test and Fisher’s exact test for categorical variables. Receiver operating characteristics (ROC) curves were established to determine the optimal cut-off value for AKI based on the Youden index. Multivariate logistic regression was used to analyse the risk factors for AKI and multivariate Cox regression was used to analyse the risk factors for kidney relapse. Forward likelihood ratios by partial likelihood estimators were used in the above multivariate analysis. Kaplan–Meier survival curves were used to analyse the time to CRR and relapse in patients with lupus podocytopathy. All statistical tests were bilateral and *P* < .05 was considered statistically significant.

## RESULTS

### Baseline characteristics

Among the 6052 LN patients, 98 (1.6%) were diagnosed with lupus podocytopathy. Of these, 85 were female and 13 were male and they had a median age of 29 years and a median SLEDAI-2K score of 9 points. Baseline clinical, pathological and laboratory data are presented in Table 1.

**Table 1:** Clinical, laboratory and pathologic findings of the patients with lupus podocytopathy.

		Comparison		
Parameters	Total (*N* = 98)	MGL (*n* = 71)	FSGS (*n* = 27)	*P*-value
Age (years)	29 (22–42)	31 (21–41)	28 (23–43)	.98
Male/female, *n*/*n*	13/85	8/63	5/22	.34
SLE duration (months)	3 (1–21)	3 (1–15)	4 (1–29)	.94
Duration of kidney disease (months)	1 (1–6)	1 (1–8)	1 (1–5)	.55
SLEDAI-2K score	9 (5–12)	8 (4–12)	10 (8–12)	.04
Renal SLEDAI score	4 (4–8)	4 (4–4)	4 (4–8)	.03
Hypertension, *n* (%)	24 (24.5)	13 (18.3)	11 (40.7)	.03
Microscopic haematuria, *n* (%)	21 (21.4)	11 (15.5)	10 (37.0)	.03
Proteinuria (g/24 h)	6.78 (4.50–10.00)	6.00 (4.50–9.51)	7.82 (4.60–11.96)	.11
Serum albumin (g/l)	20.6 (18.9–26.0)	20.8 (19.3–25.6)	19.9 (17.2–26.3)	.44
SCr (mg/dl)	0.78 (0.65–1.36)	0.74 (0.64–1.15)	0.91 (0.66–2.21)	.18
AKI, *n* (%)	34 (34.7)	23 (32.4)	11 (40.7)	.48
ANA positive, *n* (%)	98 (100.0)	76 (100.0)	26 (100.0)	ND
Anti-dsDNA positive, *n* (%)	29 (29.6)	19 (26.8)	10 (37.0)	.33
APL positive, *n* (%)	37 (37.8)	27 (38.0)	10 (37.0)	>.99
Serum C3 (g/l)	0.677 (0.496–0.845)	0.690 (0.478–0.845)	0.634 (0.500–0.820)	.68
Serum C4 (g/l)	0.135 (0.090–0.192)	0.134 (0.089–0.186)	0.135 (0.100–0.196)	.86
Extrarenal involvement, *n* (%)				
Haematology	51 (52.0)	36 (50.7)	15 (55.6)	.82
Skin	47 (48.0)	37 (52.1)	10 (37.0)	.26
Joint	31 (31.6)	22 (31.0)	9 (33.3)	>.99
CNS	16 (16.3)	14 (19.7)	2 (7.4)	.22
Intestine	15 (15.3)	8 (11.3)	7 (25.9)	.11
Heart	15 (15.3)	9 (12.7)	6 (22.2)	.35
Pathologic changes				
FPE (%)	75 (65–85)	75 (60–85)	85 (65–90)	.11
ATI score	1 (0–2)	0 (0–1)	1 (0–2)	.03
CTI score	0 (0–1)	0 (0–0)	1 (0–1)	<.001
Mesangial immune deposits, *n* (%)	88 (89.8)	65 (91.5)	23 (85.2)	.46
Tubular immune deposits, *n* (%)	20 (20.4)	14 (19.7)	6 (22.2)	>.99
Full-house pattern, *n* (%)	39 (39.8)	32 (45.1)	7 (25.9)	.107

Values are presented as median (IQR) unless stated otherwise.

ND: not done; APL: anti-phospholipid antibodies; CNS: central nervous system.

All patients exhibited NS with a median urinary protein excretion of 6.78 g/24 h (IQR 3.12–8.47) and 21 (21.4%) of them had microscopic haematuria. There were 34 patients (34.7%) experiencing AKI, with 6 (6.1%) requiring renal replacement therapy. Extrarenal involvement included haematology (52.0%), skin (48.0%), joints (31.6%), central nervous system (16.3%), intestine (15.3%) and heart (15.3%).

Kidney biopsy showed MGL in 71 patients (72.4%) and FSGS in 27 patients (27.6%). The rates of hypertension and microscopic haematuria and the SLEDAI-2K, renal SLEDAI, ATI and CTI scores in the FSGS group were significantly higher than those in the MGL group. The AKI incidence and SCr level in the FSGS group were higher than those in the MGL group, but the differences were not statistically significant. There were no statistical differences in the severity of proteinuria, immune indices, extrarenal organ injury, FPE and deposition in the mesangial area between the two groups.

### Risk factors for AKI

Patients with lupus podocytopathy were divided into the AKI group (*n* = 34) and non-AKI group (*n* = 64) according to the AKI diagnostic criteria. Compared with patients in the non-AKI group, patients in the AKI group showed much higher levels of proteinuria, ATI score and FPE but significantly lower levels of serum albumin and C3 (Table 2).

**Table 2:** Comparisons of clinical and pathologic findings in lupus podocytopathy patients in the AKI and non-AKI groups.

Parameters	AKI group (*n* = 34)	Non-AKI group (*n* = 64)	*P*-value
Age (years)	32 (22–44)	28 (21–40)	.31
Female/male, *n*/*n*	7/27	6/58	.13
SLE duration (months)	4 (1–24)	2 (1–15)	.97
Duration of kidney disease (months)	1 (1–5)	2 (1–8)	.22
SLEDAI-2K	9 (7–12)	9 (4–12)	.16
Hypertension, *n* (%)	9 (26.5)	15 (23.4)	.81
Microscopic haematuria, *n* (%)	9 (26.5)	12 (18.8)	.44
Hypertension (g/24 h)	9.56 (7.29–11.76)	5.41 (3.88–7.96)	<.001
Serum albumin (g/l)	19.7 (18.0–22.9)	23.5 (19.3–28.5)	<.001
SCr (mg/dl)	2.12 (1.30–4.07)	0.67 (0.57–0.79)	<.001
ANA positive, *n* (%)	34 (100.0)	64 (100.0)	ND
Anti-dsDNA positive, *n* (%)	10 (29.4)	19 (29.7)	>.99
APL positive, *n* (%)	8 (23.5)	24 (37.5)	.18
Serum C3 (g/l)	0.583 (0.415–0.740)	0.763 (0.546–0.879)	.005
Serum C4 (g/l)	0.122 (0.059–0.173)	0.138 (0.100–0.195)	.29
Pathologic changes			
FPE (%)	85 (74–90)	75 (55–85)	.04
ATI	2 (1–2)	0 (0–1)	<.001
CTI	0 (0–0)	0 (0–1)	.63
Mesangial immune deposition, *n* (%)	32 (94.1)	56 (63.6)	.26
Tubular immune deposition, *n* (%)	11 (32.4)	9 (14.1)	.04

Values are presented as median (IQR) unless stated otherwise.

ND: not done; APL: anti-phospholipid antibodies; CNS: central nervous system.

**Figure 2:**
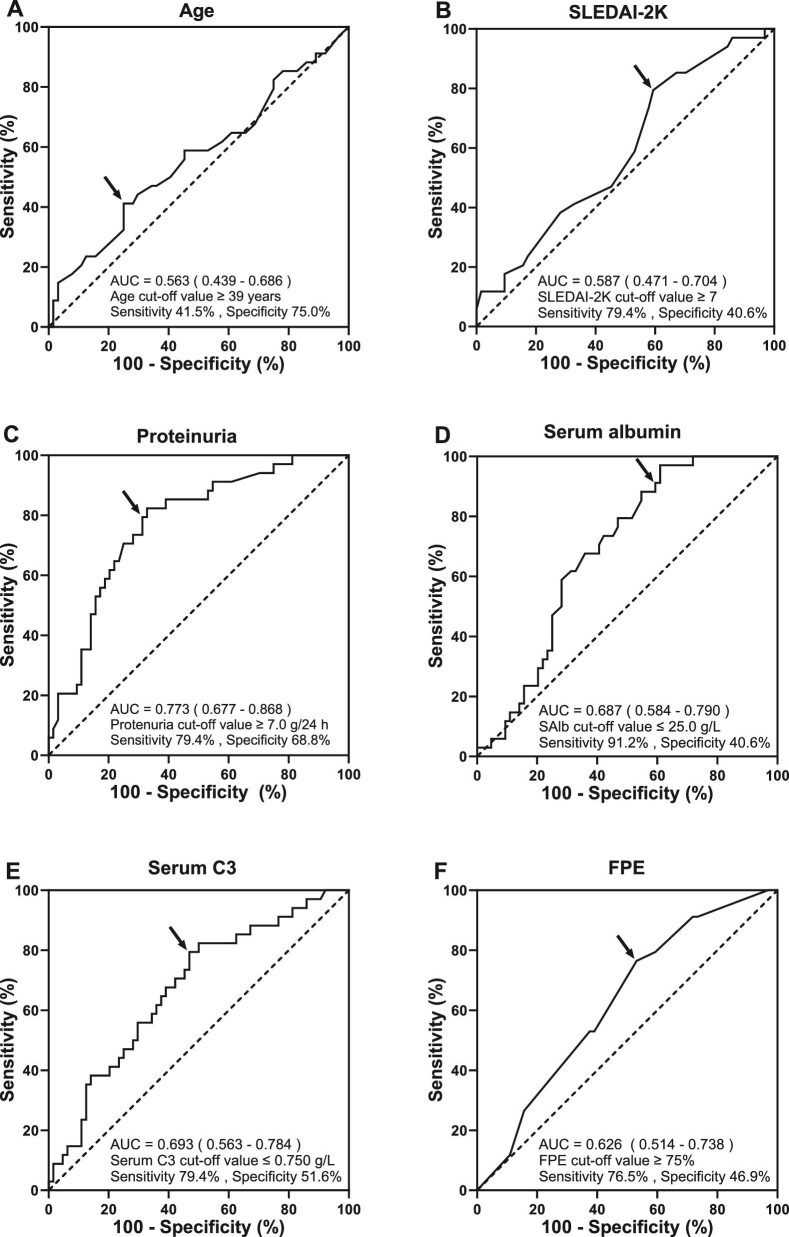
ROC curve for determining the optimal cut-off value for AKI development in patients with lupus podocytopathy. AUC: area under the curve.

The optimal cut-off values for AKI were determined according to ROC curves (Fig. 2). Multivariate logistic regression analysis revealed that proteinuria ≥7.0 g/24 h [odds ratio (OR) 6.120 (95% CI 1.848–20.265), *P* = .003] and serum C3 ≤0.750 g/l [OR 6.842 (95% CI 2.221–21.071), *P* = .001] were independent risk factors for AKI (Table 3).

**Table 3:** Univariate and multivariate logistic regression analyses of risk factors for AKI in patients with lupus podocytopathy.

	Univariate analysis		Multivariate analysis	
Variables	OR (95% CI)	*P*-value	OR (95% CI)	*P*-value
Sex				
Female	0.399 (0.122–1.301)	.13	NS	
Male	Reference			
Age (years)^a^				
≥39	2.100 (0.865–5.098)	.10	NS	
<39	Reference			
SLEDAI-2K^a^				
≥7	2.639 (1.001–6.958)	.05	NS	
<7	Reference			
Proteinuria^a^				
≥7.0 g/24 h	8.486 (3.169–22.726)	<.001	6.120 (1.848–20.265)	.003
<7.0 g/24 h	Reference		Reference	
Serum albumin^a^				
≤25.0 g/l	7.070 (1.954–25.576)	.003	NS	
>25.0 g/l	Reference			
Anti-dsDNA				
Positive	0.987 (0.396–2.457)	.98	NS	
Negative	Reference			
Serum C3^a^,^b^				
≤0.750 g/l	4.106 (1.564–10.778)	.003	6.842 (2.221–21.071)	.001
>0.750 g/l	Reference		Reference	
Glomerular lesions				
FSGS	1.435 (0.575–3.581)	.44	NS	
MGL	Reference			
FPE^a^				
≥75%	2.868 (1.129–7.284)	0.03	NS	
<75%	Reference			

NS: not statistically significant in multivariate analysis.

^a^Cut-off value obtained from ROC curve by Youden index.

^b^The normal range of serum C3 is 0.8–1.8 g/l.

### Treatment response

**Figure 3:**
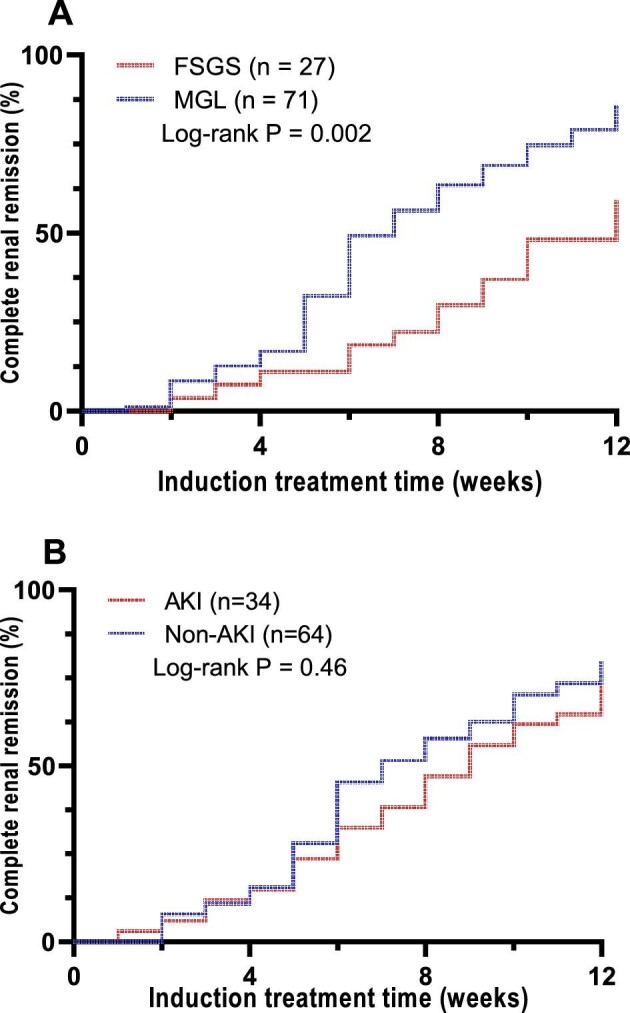
Cumulative complete renal response rate within 12 weeks after induction treatment. **(A)** The comparison between glomerular MGL and FSGS lesions. **(B)** The comparison between AKI and non-AKI groups.

Fifty-nine patients received glucocorticoid monotherapy and the remaining 39 received combination therapy for induction treatment. Within the 12 weeks of induction treatment, renal remission was achieved in 93 patients (94.9%), including 77 (78.6%) with CRR and 16 (16.3%) with PRR, and the median time to achieve renal remission was 5 weeks (IQR 4–7) (Table 4). There was no difference in the CRR rate between the glucocorticoid monotherapy and combination induction regimens (76.3% versus 82.1%; *P* = .62). The CRR rate within 12 weeks of treatment was significantly lower in the FSGS group than in the MGL group (59.3% versus 85.9%; *P* = .006). Survival analysis revealed a significantly lower cumulative CRR rate in the FSGS group compared with the MGL group, while no statistical difference was observed between the AKI and non-AKI groups (Fig. 3).

**Table 4:** Treatment responses and kidney outcomes in the MGL and FSGS groups of lupus podocytopathy.

		Comparison		
Treatment response and kidney outcome	Total (*n* = 98)	MGL (*n* = 71)	FSGS (*n* = 27)	*P*-value
Induction with GC monotherapy, *n* (%)	59 (60.2)	46 (64.8)	13 (48.1)	.17
Induction with combination therapy, *n* (%)	39 (39.8)	25 (35.2)	14 (51.9)	.17
Treatment response within 12 weeks, *n* (%)				.005^a^
Complete renal remission	77 (78.6)	61 (85.9)	16 (59.3)	
Partial renal remission	16 (16.3)	9 (12.7)	7 (25.9)	
No response	5 (5.1)	1 (1.4)	4 (14.8)	
Time to remission (weeks), median (IQR)	5 (4, 7)	5 (4, 7)	5 (3, 9)	.85
Maintenance with GC monotherapy, *n* (%)	15 (15.8)	12 (16.9)	3 (12.5)	.75
Maintenance with combination therapy, *n* (%)	80 (84.2)	59 (83.1)	21 (87.5)	.75
Follow-up time (months), median (IQR)	78 (35, 134)	89 (59, 155)	33 (9, 89)	<.001
Kidney relapse, *n* (%)^b^	57 (60.0)	43 (60.6)	14 (58.3)	>.99

^a^Data were analysed as ordinal data.

^b^95 patients achieved renal remission during follow-up.

**Figure 4:**
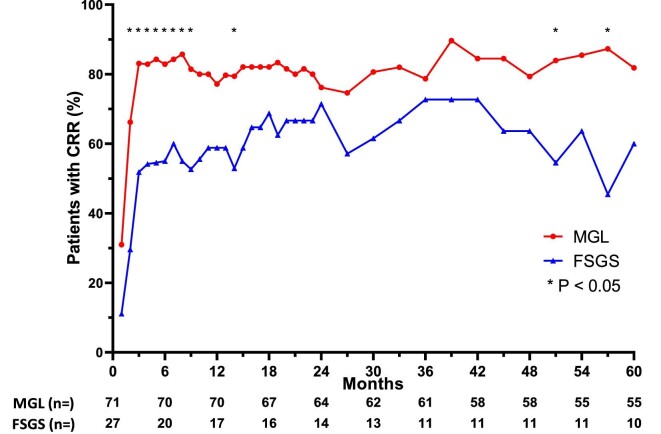
CRR over time between the MGL and FSGS groups of patients with lupus podocytopathy.

Three patients who did not achieve renal remission after 12 weeks of treatment were lost to follow-up. During a median follow-up of 78 months (IQR 35–134), a total of 95 patients (96.9%) achieved renal remission. Compared with those in the FSGS group, patients in the MGL group had significantly higher CRR rates at 2–9, 14, 51 and 57 months of follow-up (Fig. 4).

### Kidney relapse and outcome

Among the 95 patients who achieved renal remission during the follow-up period, kidney relapse occurred in 57 patients (60.0%) (the relapse group) but not in the remaining 38 (the non-relapse group). Compared with the non-relapse group, the relapse group had significantly higher SLE duration, SCr level, AKI and anti-double-stranded DNA (dsDNA)-positive rates, ATI score and glucocorticoid monotherapy rate but a significantly lower CRR rate within 12 weeks (92.1% versus 73.7%; *P* = .03; Table 5).

**Table 5:** Comparisons of clinical and pathologic findings, treatment responses and kidney outcomes between the lupus podocytopathy patients in the relapse group and non-relapse group.

Parameters	Relapse group (*n* = 57)	Non-relapse group (*n* = 38)	*P*-value
Age (years)	31 (23–43)	28 (19–38)	.35
Male/female, *n*/*n*	6/51	6/32	.53
SLE duration 9months)	6 (1–24)	2 (1–10)	.05
Duration of renal disease (months)	2 (1–11)	1 (1–2)	.17
SLEDAI-2K	9 (4–12)	9 (6–12)	.69
Hypertension, *n* (%)	16 (28.1)	6 (15.8)	.22
Microscopic haematuria, *n* (%)	10 (17.5)	10 (26.3)	.44
Hypertension (g/24 h)	6.93 (4.50–10.00)	5.69 (4.13–9.08)	.19
Serum albumin (g/l)	20.8 (19.3–25.7)	21.2 (18.7–27.8)	.51
SCr (mg/dl)	0.84 (0.67–2.20)	0.69 (0.57–0.94)	.008
AKI, *n* (%)	25 (43.9)	8 (21.1)	.03
ANA positive, *n* (%)	57 (100)	38 (100)	ND
Anti-dsDNA positive, *n* (%)	12 (21.1)	16 (42.1)	.04
APL positive, *n* (%)	19 (33.3)	13 (34.0)	>.99
Serum C3 (g/l)	0.690 (0.493–0.829)	0.638 (0.503–0.857)	.86
Serum C4 (g/l)	0.151 (0.095–0.200)	0.126 (0.073–0.157)	.33
Pathologic changes			
FPE (%)	85 (65–85)	70 (64–85)	.11
ATI score	1 (0–2)	0 (0–1)	.03
CTI score	0 (0–1)	0 (0–0)	.29
Mesangial immune deposition, *n* (%)	50 (87.7)	35 (92.1)	.74
Tubular immune deposition, *n* (%)	11 (19.3)	7 (18.4)	>.99
Induction with GC monotherapy, *n* (%)	34 (59.6)	23 (60.5)	>.99
Induction with combination therapy, *n* (%)	23 (40.4)	15 (39.5)	>.99
Treatment response within 12 weeks, *n* (%)			.03
Complete renal remission	42 (73.7)	35 (92.1)	
Partial renal remission	15 (26.3)	3 (7.9)	
Time to remission (weeks)	5 (5–7)	5 (3–7)	.26
Maintenance with GC monotherapy, *n* (%)	13 (22.8)	2 (5.3)	.02
Maintenance with combination therapy, *n* (%)	44 (77.2)	36 (94.7)	.02

Values are presented as median (IQR) unless stated otherwise.

ND: not done; APL: anti-phospholipid antibodies; CNS: central nervous system.

**Figure 5.**
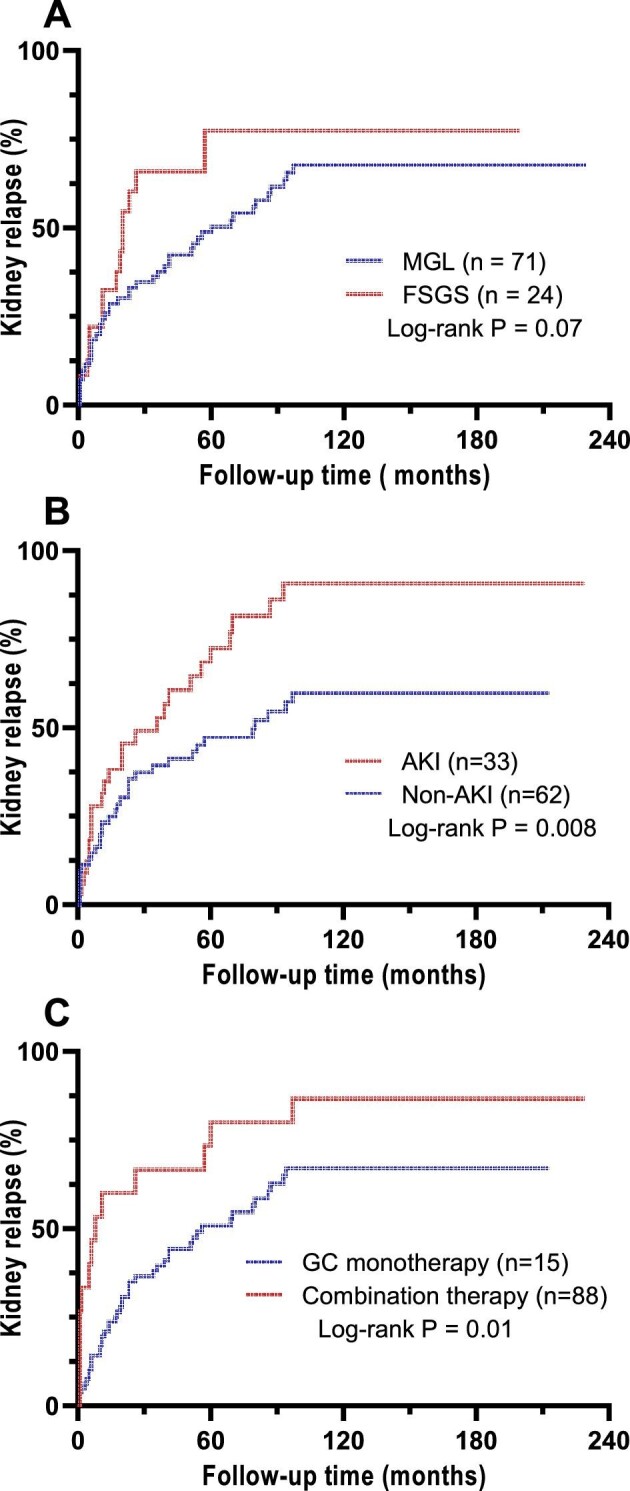
Cumulative kidney relapse rate during follow-up. **(A)** The comparison between glomerular MGL and FSGS lesions. **(B)** The comparison between the AKI and non-AKI groups. **(C)** The comparison between patients receiving glucocorticoid monotherapy and those receiving combination therapy.

Multivariate Cox regression analysis showed that failure to achieve CRR within 12 weeks [HR 3.595 (95% CI 1.928–6.703), *P* < .001], glucocorticoid monotherapy for maintenance treatment [HR 2.092 (95% CI 1.123–3.895), *P* = .02] and AKI at onset [HR 1.842 (95% CI 1.085–3.128), *P* = .02] were independent risk factors for kidney relapse in patients with lupus podocytopathy (Table 6). Survival analysis showed no statistical difference in cumulative kidney relapse rates between the FSGS and MGL groups, while the cumulative kidney relapse rate in the AKI group was significantly higher than that in the non-AKI group. Patients who received glucocorticoid monotherapy had significantly higher renal relapse rate than those who received combination therapy (Fig. 5).

**Table 6:** Univariate and multivariate Cox proportional hazards regression analysis of risk factors for kidney relapse in patients with lupus podocytopathy.

	Univariate analysis		Multivariate analysis	
Variables	HR (95% CI)	*P*-value	HR (95% CI)	*P*-value
Age (years)	1.013 (0.992–1.034)	.22	NS	
Female	1.251 (0.536–2.920)	.60	NS	
SLE duration (months)	1.012 (0.997–1.027)	.11	NS	
SLEDAI-2K score	0.997 (0.949–1.048)	.90	NS	
AKI	2.006 (1.179–3.411)	.01	1.842 (1.085–3.128)	.02
Proteinuria (g/24 h)	1.063 (1.005–1.125)	.03	NS	
Serum albumin (g/l)	0.967 (0.921–1.014)	.17	NS	
Anti-dsDNA positive	0.561 (0.296–1.061)	.08	NS	
Serum C3 (g/l)	1.033 (0.463–2.306)	.94	NS	
Glomerular FSGS lesion	1.741 (0.934–3.245)	.08	NS	
ATI	1.400 (1.059–1.849)	.02	NS	
FPE	1.010 (0.991–1.029)	.32	NS	
Not CRR within 12 weeks	3.789 (2.034–7.059)	<.001	3.595 (1.928–6.703)	<.001
Maintenance with GC monotherapy	2.128 (1.144–3.958)	.02	2.092 (1.123–3.895)	.02

NS: not statistically significant in multivariate analysis.

No patient died or reached the kidney endpoint during the follow-up period. Twenty patients underwent repeat kidney biopsy after a median of three kidney relapses and histological transformation was observed in 10 patients, including 6 patients transforming to class V, 3 to class IV and 1 to class IV + V.

## DISCUSSION

This retrospective cohort study of lupus podocytopathy had the largest sample size to date and found that lupus podocytopathy patients constituted 1.6% of the LN patients with kidney biopsy, which was in agreement with previous studies [2, 4, 5, 10]. We also found that more than one-third of lupus podocytopathy patients presented with AKI. Most of the patients with lupus podocytopathy showed rapid response to induction treatment, but nearly 60% of them experienced kidney relapse. Logistic regression results confirmed that the levels of proteinuria and serum C3 were correlated with AKI in the patients with lupus podocytopathy. Multivariate Cox regression analysis showed that AKI, treatment response and maintenance treatment regimen were the major risk factors associated with kidney relapse.

Lupus podocytopathy had three glomerular lesions of morphology as described in previous studies, minimal change disease, mesangial proliferation and FSGS. However, no differences were observed in clinical features and treatment response between the patients with minimal change disease and those with mesangial proliferation [3, 10]. Consequently, we combined these two subtypes as MGL group, which exhibited distinct clinical and pathological characteristics and treatment response from the FSGS group.

In our cohort, the MGL subtype accounted for the majority of patients with lupus podocytopathy, while only 30% of patients were of the FSGS subtype. The differences between the two groups are as follows. First, the rates of hypertension and microscopic haematuria, the levels of SLE activity and the score of renal tissue injury in the FSGS group were significantly higher than in the MGL group. Our previous study reported that the FSGS group had a higher rate of AKI compared with the MGL group [10]. In this study, the rate of AKI and the level of SCr in the FSGS group were higher than in the MGL group, although the differences were not statistically significant, which suggested that kidney injury in the FSGS group was more severe than in the MGL group. Second, patients with an FSGS lesion showed poor treatment response, as shown by the fact that the CRR rate within 12 weeks in the FSGS group was significantly lower than in the MGL group, and this difference remained in the follow-up period. Therefore, patients with an FSGS lesion require an optimal treatment regimen to improve the early CRR rate. Finally, FSGS and minimal change disease were considered as different stages of primary podocytopathy [16]. In our study, all patients in the FSGS group underwent renal biopsy at the onset of LN. There was only one patient in the MGL group switched to the FSGS group after relapse according to the repeat renal biopsy. However, it could not be ruled out that this patient might have had early FSGS lesion that was missed due to the limited number of glomeruli examined in the first biopsy section. Therefore, our current data do not support the notion that FSGS is transformed from MGL, but do suggest that these two subtypes of lupus podocytopathy are pathologically distinct.

Our study confirmed the presence of acute tubulointerstitial injury in lupus podocytopathy patients with AKI. In addition, for the first time, we discovered that high proteinuria and low serum C3 levels are correlated with AKI in patients with lupus podocytopathy. Proteinuria is considered a risk factor for AKI in glomerular diseases [17]. The overloaded urinary protein can result in excessive production of reactive oxygen species and significantly increased expression of inflammatory cytokines and chemokines, leading to tubular injury [18–22]. Obstruction of tubular urinary protein casts, collapse of renal tubules caused by interstitial oedema and renal vasoconstriction caused by an abnormal increase of endothelin-1 are also considered as the possible pathogenesis for AKI [23, 24]. The high proportion of tubular immune complex deposition in patients with AKI suggests that complement activation at the renal tubular epithelium is an important mechanism of tubule injury in lupus podocytopathy. The surface of epithelial cells is more sensitive to membrane attack complex due to a lack of membrane-bound complement regulatory factors [25]. Various components of complements in urine may be activated on the surface of epithelial cells after podocyte injury, resulting in tubular injury [26].

Kidney relapse is common in patients with lupus podocytopathy and a proportion of patients may experience repeated kidney relapses, resulting in an increased risk of adverse reactions from exposure to high doses of glucocorticoids and other immunosuppressants [27]. Our study is the first to find that kidney function at onset, early treatment response and the maintenance treatment regimen are independent risk factors for kidney relapse in patients with lupus podocytopathy. It is difficult for patients who receive glucocorticoid monotherapy to maintain a remission state, as reported in our previous study [10]. During maintenance treatment, the combination of glucocorticoids and immunosuppressants, such as anti-CD20 or anti-B cell activating factor monoclonal antibody can potentially improve kidney relapse [28].

In the present study, no patient died or reached the kidney endpoint during the follow-up period. It may be due to the high CRR rates, kidney functional recovery after treatment (even in patients with kidney relapse) and the low rate of extrarenal organ injuries (especially involvement of the heart and central nervous system) in lupus podocytopathy patients.

Although our present study was conducted in a large cohort of lupus podocytopathy patients, there were still several limitations. First, this was a single-centre, retrospective study that needs to be further verified by multicentre studies. Second, urine AKI markers were not included in our study due to limited data accessibility. Third, the treatment regimen varied for individual patients, making it difficult to compare the treatment response between different immunosuppressive regimens.

In conclusion, our study revealed the incidence of lupus podocytopathy in LN and the different treatment responses between patients with MGL and FSGS. This study also suggests that an optimal treatment regimen is required to improve the renal remission rate in patients with FSGS. Finally, for the first time, our study identified the independent risk factors for AKI and kidney relapse in patients with lupus podocytopathy.

## ACKNOWLEDGEMENTS

The kidney biopsy samples in this study were from the Kidney Biobank of the National Clinical Research Center of Kidney Diseases, Jinling Hospital, Nanjing, China.

## Data Availability

The data underlying this article will be shared upon reasonable request to the corresponding author.
